# Multivariate modeling to identify patterns in clinical data: the example of chest pain

**DOI:** 10.1186/1471-2288-11-155

**Published:** 2011-11-22

**Authors:** Oliver Hirsch, Stefan Bösner, Eyke Hüllermeier, Robin Senge, Krzysztof Dembczynski, Norbert Donner-Banzhoff

**Affiliations:** 1 Department of General Practice/Family Medicine, Philipps University Marburg, Germany; 2 Department of Mathematics and Computer Science, Knowledge Engineering & Bioinformatics, Philipps University Marburg, Germany

## Abstract

**Background:**

In chest pain, physicians are confronted with numerous interrelationships between symptoms and with evidence for or against classifying a patient into different diagnostic categories. The aim of our study was to find natural groups of patients on the basis of risk factors, history and clinical examination data which should then be validated with patients' final diagnoses.

**Methods:**

We conducted a cross-sectional diagnostic study in 74 primary care practices to establish the validity of symptoms and findings for the diagnosis of coronary heart disease. A total of 1199 patients above age 35 presenting with chest pain were included in the study. General practitioners took a standardized history and performed a physical examination. They also recorded their preliminary diagnoses, investigations and management related to the patient's chest pain. We used multiple correspondence analysis (MCA) to examine associations on variable level, and multidimensional scaling (MDS), k-means and fuzzy cluster analyses to search for subgroups on patient level. We further used heatmaps to graphically illustrate the results.

**Results:**

A multiple correspondence analysis supported our data collection strategy on variable level. Six factors emerged from this analysis: „chest wall syndrome“, „vital threat“, „stomach and bowel pain“, „angina pectoris“, „chest infection syndrome“, and „ self-limiting chest pain“. MDS, k-means and fuzzy cluster analysis on patient level were not able to find distinct groups. The resulting cluster solutions were not interpretable and had insufficient statistical quality criteria.

**Conclusions:**

Chest pain is a heterogeneous clinical category with no coherent associations between signs and symptoms on patient level.

## Background

People rarely experience one symptom in isolation. Physicians are confronted with numerous interrelationships between symptoms and with evidence for or against classifying a patient into a diagnostic category. They categorize patients with regard to diagnosis, prognosis, and/or further management. Therefore, finding groups in patient data is important in several respects. One might identify subgroups of patients who can benefit from different interventions. Certain symptom configurations might be a cue for further investigations [1]. Diagnostic categories have developed over time, by tradition or by gaining pathophysiological insights. Feedback about the adequacy of such categories often occurs on the individual patient level and is therefore non-systematic. We rarely found systematic studies which successfully applied multivariate models to information available to clinicians. Therefore, it might be worthwhile to model clinicians' decision making by multivariate techniques.

Coronary heart disease (CHD) is one of the main underlying aetiologies for chest pain in primary care [2]. Primary care practitioners start the diagnostic process with the history and physical examination [3] and order further examinations like as needed [4]. Physicians hereby combine the information gained from the above diagnostic tests [3,5].

Statistical measures for grouping variables or patients have rarely been applied to chest pain data, mostly in connection with finding subgroups in acute coronary heart disease. We only found one study in chest pain which performed a k-means cluster analysis on variable level to determine whether there are characteristic patterns in chest pain locations described by patients [6]. The grouping of four different chest pain locations on variable level were not helpful in differentiating between patients with an acute coronary syndrome (ACS) and patients with non-cardiac chest pain. Studies with patients with confirmed cardiac diagnoses were also not able to find characteristic symptom patterns within these diagnostic groups by using cluster analytic techniques [1,7-9].

We intended to use statistical methods to identify natural groups of patients with chest pain on the basis of a larger, comprehensive data set with information on history, risk factors, and physical examination [10,11]. These groups should then be validated with patients' diagnoses in order to see if they have predictive power. The applied multivariate techniques enable us to handle a large amount of data in order to unfold hidden patterns. This strategy was not followed previously in other studies.

Patients were enrolled consecutively in a primary care setting so that patient recruitment was not distorted by referral patterns. This could have been the case if we had included specialized physicians (e.g. cardiologists) who get a selection of patients referred to them by primary care doctors.

## Methods

We conducted a cross-sectional diagnostic study in a primary care setting [12] with the primary aim to establish the validity of symptoms and findings for the diagnosis of coronary heart disease [10,11]. The study protocol was approved by the ethics committee of the Faculty of Medicine, University of Marburg. The study complies with the Declaration of Helsinki. Here, we present a secondary analysis to find natural groups in our data.

### Participating GPs and patients

We approached 209 general practitioners (GPs) in the Land of Hesse of whom 74 (35.4%) agreed to participate in the study. Participating practices had to recruit consecutively every attending patient who had chest pain. The recruitment period lasted 12 weeks for each practice.

Every patient above 35 years with chest pain was to be included. Patients were eligible irrespective of the acute or chronic nature of their complaint, or of previously known conditions including CHD or risk factors. Patients whose chest pain had subsided for more than one month, whose chest pain had been investigated already and/or who came for follow-up for chest pain were excluded. This resulted in a total of 1199 patients.

### Data collection

The data collection strategy was based on the current literature on chest pain and on feedback by GPs [13]. The GPs took a standardized history and performed a physical examination. They also recorded their preliminary diagnoses, investigations and management related to the patient's chest pain. Patients were contacted by phone six weeks and six months after the index consultation. Study assistants blinded to clinical data already recorded asked about the course of their chest pain, treatments including hospitalisations and drugs. Discharge letters from specialists and hospitals were requested from GPs.

Random audits were performed searching routine documentation of participating practices to identify cases of chest pain not included in the study.

After 6 months a reference panel consisting of one cardiologist, one GP and one research staff of the department of General Practice/Family Medicine decided about the presumed diagnosis at the time of patient recruitment on the basis of the GP's documentation and follow up data.

### Statistical Methods

For statistical grouping techniques like cluster analysis it is important that the underlying constructs are reliably measured and relevant variables are included in the analyses [14]. Consequently, we first examined whether the content of our case report form was capable of capturing relevant aspects associated with chest pain. A conventional factor analysis was not feasible because of the binary scaling of our data. Therefore, we performed a multiple correspondence analysis (MCA) on variable level with all variables of our case report form. We only excluded those variables with extremely skewed distributions. MCA is an exploratory method which performs cross tabulations of all included nonmetric variables [15]. A virtually unlimited number of variables can be included in this analysis. All variables in a data set can be related to each other without differentiation between descriptive and explanatory variables. In contrast to other multivariate measures, MCA does not require large sample sizes and can even be calculated with n = 10 [16]. Recently, MCA was considered being a useful tool to uncover the relationships among categorical variables [17]. We chose the factor analytic interpretation of MCA because of our high number of variables. Calculations were done using ALMO 12 (http://www.almo-statistik.de).

Based on the results of MCA, we applied two visualization techniques in order to get a first idea of any specific structure in the data, notably in terms of a natural clustering into subgroups. We used multidimensional scaling (MDS) and hierarchical clustering combined with a so-called heat map representation. Both techniques are implemented in the R package *stats *(http://www.R-project.org).

The first method, MDS, seeks to embed the original observations as vectors in a low-dimensional space, typically the two-dimensional Euclidean space, so as to preserve the pairwise distances between these observations as far as possible (formally, the problem is stated as minimizing the sum of squared differences between the original dissimilarities and the Euclidian distances in the low-dimensional space). Thus, MDS only requires dissimilarities between observations as input. If data are given as multidimensional vectors, the dissimilarities can be computed as Euclidean distances between these vectors. If natural clusters exist within the data, these clusters should also become visible in the low-dimensional representation of the data [18].

A heat map represents the pairwise distance (similarity) matrix in terms of an image, with distance (similarity) degrees indicated by colours (similarities can be computed as reciprocals of the Euclidean distance between observations): The darker the colour, the more similar the observations are. Prior to printing the image, the sequence of observations is reordered in such a way that similar observations tend to be neighboured. More specifically, this reordering is determined by means of a hierarchical clustering of the data, and the corresponding dendrogram is added to the left and to the top side of the image. Since the similarity matrix is symmetric, both dendrograms are the same, and the image itself is symmetric, too. Clusters within the data become visible as "warm-shaded squares" along the diagonal of the heat map.

In a next step, we performed k-means cluster analyses on patient level, generalized to all scales of measurement with weighted squared euclidean distances [19,20]. The k-means procedure identifies relatively homogenous subgroups while maximizing the variability between clusters and is able to handle larger amounts of classification variables. Variables with mixed scaling can be handled in this approach [19,21,22]. Calculations were done with ALMO 12 which includes a k-means algorithm that is able to handle the different scaling of our variables and the large sample size. Schendera [21] states that a sample size of n = 250 is too large for some cluster analysis algorithms. This programme provides statistical measures to evaluate the appropriate number of clusters and the model fit. For this, an F value and eta^2 ^for each cluster solution is calculated that indicate the contribution of the classification variables to the separation of the clusters. One may then choose the solution with the highest F value and largest explained variance (eta^2^). If this solution is not reasonably interpretable with regards to content, then it is admissible to choose another solution [19].

To account for a possible fuzziness in our data, we additionally undertook fuzzy cluster analyses with the programme NCSS2007 (http://www.ncss.com) [14]. In common cluster analysis, each patient is assigned to only one cluster. In fuzzy clustering, a patient can be partially classified into more than one cluster. Goodness of fit of a fuzzy clustering solution can be assessed by the normalized Dunn partition coefficient Fc(U) that ranges from 0 (completely fuzzy) to 1 (hard clustering) and by the coefficient Dc(U) which ranges from 0 (hard clustering) to 1-(1/K) (completely fuzzy), where K is the number of clusters. The number of clusters should be chosen so that Fc(U) is large and Dc(U) is small [14,23]. Another goodness of fit indicator is the average silhouette per cluster. It ranges from -1 to +1. An average silhouette from 0.71 to 1.00 denotes that a strong structure has been found, an average silhouette from 0.51 to 0.70 indicates a reasonable structure, a value from 0.26 to 0.50 means that the structure is weak and could be artificial, and a value from 0.25 to -1 refers to no substantial structure [23].

## Results

### Multiple correspondence analysis (MCA) and Multidimensional scaling (MDS)

At first, by applying multiple correspondence analysis (MCA), we explored if the variables on our case report form capture relevant aspects of chest pain. We used the oblique quartimin rotation to enhance interpretability of the results. Six factors emerged from the analysis. The highest correlation between these factors is -.36 so that these factors can be regarded as being orthogonal. The eigenvalues of the six factors range from 2.08 to 3.72. The total explained variance is 24.8%. The explained variance gives a too pessimistic picture about model fit. Consequently, the fit index GFIR is recommended [19] which indicates, how many percent of the total χ^2 ^value is explained by the six factors. In our case, the GFIR shows that 57.5% of the total χ^2 ^value is explained by the six factors. This is a satisfactory result.

Table 1 lists the main results of the loading matrix that resulted from quartimin rotation. Values larger than one can occur because numbers represent coordinate points and not correlations as known from factor analysis. There are no standards to classify these loadings. They have to be put in relation to the highest loadings on each factor in order to interpret their meaning.

**Table 1:** Loading matrix after quartimin rotation of MCA data.

	F1	F2	F3	F4	F5	F6
Patient does not think that chest pain results from heart disease	**0.39**					

Chest pain at the moment of consultation	**0.50**					

Pain dependent on respiration	**1.11**					

Pain dependent on stress	**0.82**			**1.20**		

Pain dependent on exercise	**0.87**					

Localisation right	**0.54**					

No pain at palpation	**0.45**					

Pain more than once per day	**0.57**					

Patient is different than usual		**1.96**				

Something wrong with my patient.		**2.37**				

Patient is pale		**2.21**				

Patient is anxious		**0.82**				

Patient is cold sweated		**3.42**				

Patient is too quiet		**1.83**				

Patient is reddened		**1.20**				

Patient is excited		**1.01**				

Patient is short of breath		**1.88**				

Acute pain ≤48 hours		**0.82**				

Known heart failure		**0.79**				

Male gender			**0.57**			

Emesis			**0.51**			

No diabetes			**-1.31**			

No hypertension			**-0.79**			

No heart failure			**-1.24**			

No overweight			**-0.76**			

No lack of exercise			**-0.96**			

Radiation of pain into epigastrum			**0.52**			

Pressing pain				**0.61**		

Respiratory distress				**0.84**		

Tightness of the chest				**0.92**		

Radiation into left arm				**0.64**		

Duration under 30 minutes				**0.39**		

Patient is not anxious					**-0.86**	

Hollow pain					**0.60**	

Cough					**0.90**	

Respiratory infection					**1.08**	

Less frequent pain					**0.49**	

Duration of pain less than 1 minute						**0.56**

Stinging pain						**0.33**

Depicted are the highest interpretable positive and negative loadings per factor.

Factor 1 can be interpreted as „chest wall syndrome“, as it contains characteristic pain related items for this entity. Factor 2 can be named „vital threat“. It contains items typical for a life-threatening symptomatology. Factor 3 can be characterized as „stomach and bowel pain“. It consists of male patients with vomiting, radiation of pain into the upper abdomen, and with no risk factors. Factor 4 can be labelled „angina pectoris“ because of the characteristic symptoms having highest loadings on this factor. Factor 5 is interpreted as „chest infection syndrome“ and Factor 6 as „self-limiting chest pain“.

**Figure 1:**
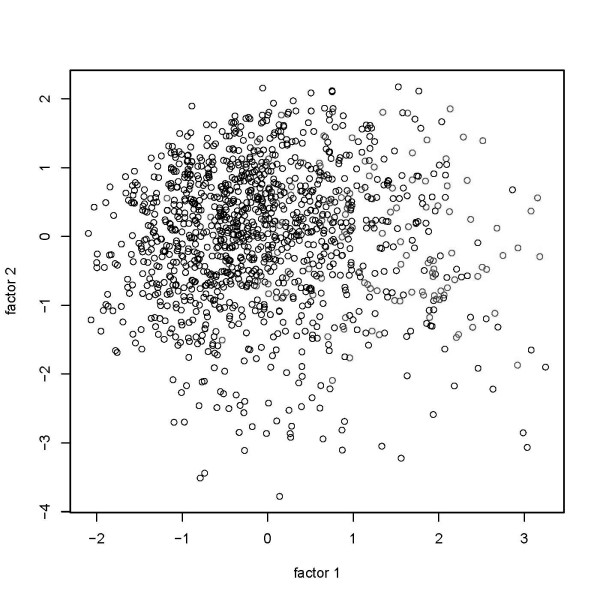
**Graphical depiction of multidimensional scaling of a two factor solution in multiple correspondence analysis**.

**Figure 2:**
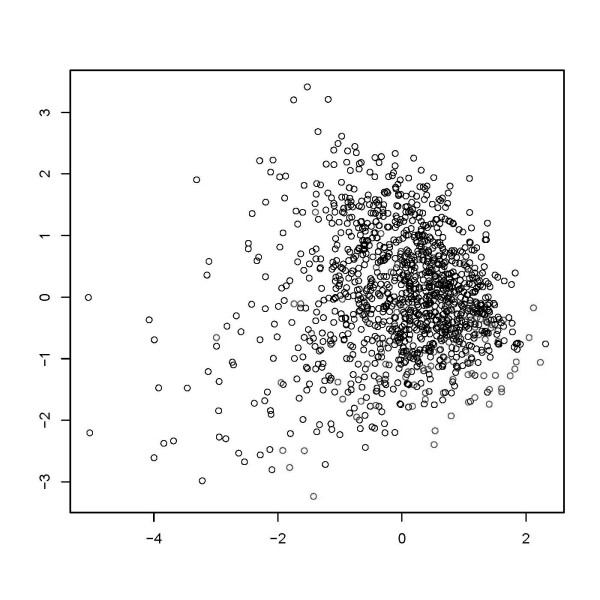
**Graphical depiction of multidimensional scaling of a six factor solution in multiple correspondence analysis**.

**Figure 3:**
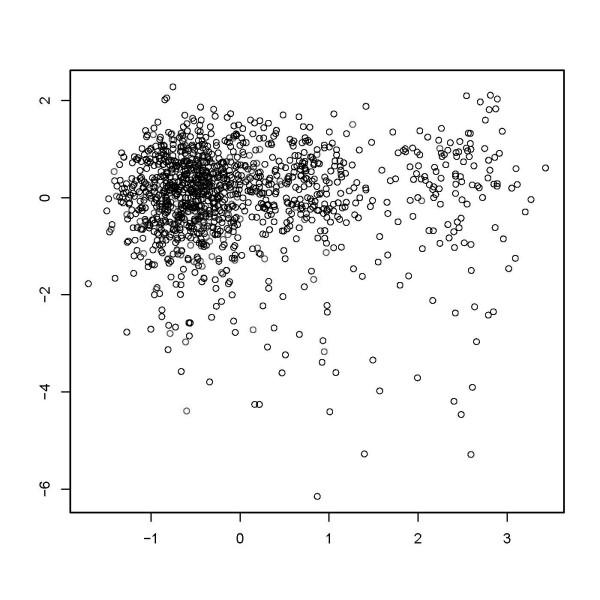
**Graphical depiction of multidimensional scaling of a twenty factor solution in multiple correspondence analysis**.

Figure 1 shows a visualization of the data points in two-dimensional space. First, we show data points in the coordinate system consisting of the first two factors obtained by MCA. Next, we show the result of a multidimensional scaling of the MCA factors to the two-dimensional plane. We used 6 and 20 factors, respectively, to compute dissimilarities (Figures 2 and 3). Obviously, a pronounced clustering structure within the data cannot be observed.

**Figure 4:**
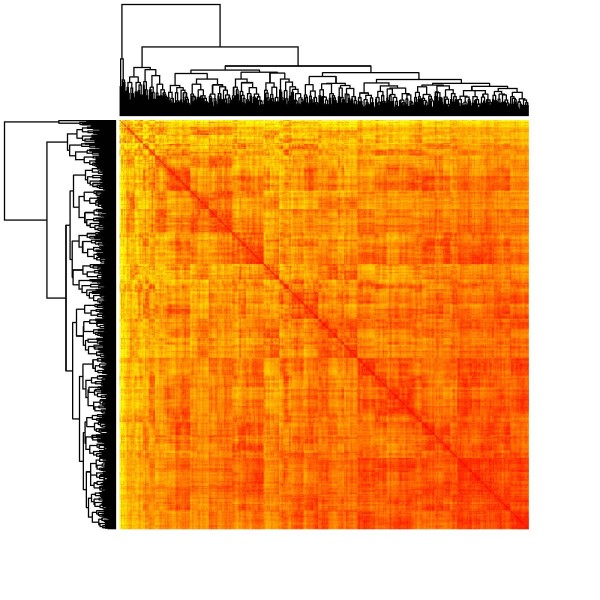
**Heatmap of the similarity matrix from hierarchical clustering on the basis of a six factor solution in multiple correspondence analysis**.

**Figure 5:**
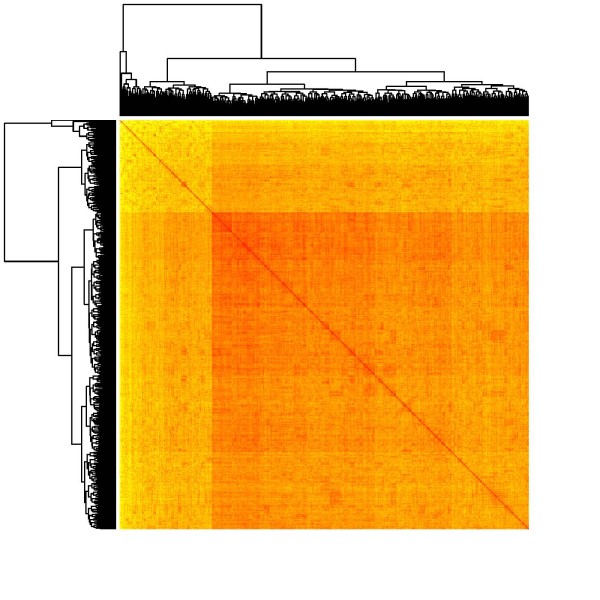
**Heatmap of the similarity matrix from hierarchical clustering on the basis of a twenty factor solution in multiple correspondence analysis**.

We also present heat maps based on the pairwise similarities between data points, using 6 and 20 factors from MCA (Figures 4 and 5). Like in the case of MDS, we were not able to observe any natural cluster structure in the data.

### Cluster analysis

A k-means cluster analysis on patient level with all variables of our case report form resulted in a three cluster solution with a small F value of 46.8 and an eta^2 ^of .073. This solution was not interpretable and had only 7.3% explained variance. Other cluster solutions were also not interpretable because of worse quality criteria.

Cluster analysis is an iterative process [19,21]. Therefore we ran several analyses and excluded variables with no meaningful contribution. The criterion was an eta^2 ^smaller .10 as a contribution of a single variable to cluster formation [24]. Even after the exclusion of irrelevant variables with regards to content and to statistical characteristics, the best quality criteria were reached at a four cluster solution with a F value of 62.2 and an eta^2 ^of .135. This solution was not interpretable and had only 13.5% explained variance. Again, other cluster solutions were also not interpretable because of worse quality criteria. The k-means approach was therefore not able to form meaningful patient groups in our data.

The best interpretable solution in fuzzy cluster analysis after excluding variables with no meaningful theoretical and numerical contribution consisted of four clusters. Cluster 1 (n = 221) can be characterized as "acute and/or threatening chest pain", Cluster 2 (n = 402) as "subacute chest pain", in Cluster 3 (n = 288), there are mainly women ≧ 65 years of age with no acute chest pain, and in Cluster 4 (n = 288), there are mainly men ≧ 55 years of age with no acute chest pain. The average silhouette of this solution was 0.54 which hardly denotes a reasonable structure in the data. The Fc(U) with .49 was low and the Dc(U) with .42 was relatively high and both indicated a high fuzziness in our data. We concluded that fuzzy clustering also was not able to partition patients into meaningful entities that have a statistical or clinical relevance.

We further cluster analyzed the factor scores on each of the six factors that resulted from our MCA. Each patient had a certain score on these six factors so that we ran a second order analysis with six variables. A k-means cluster analysis resulted in a two cluster solution with a F value of 578.2 and an eta^2 ^of .326. This solution, although statistically acceptable, was clinically not interpretable. For example, patients with vital threat and chest infection syndrome were grouped into the same cluster. Other cluster solutions with lower F values and higher number of clusters were also not clinically interpretable. The best statistical solution in fuzzy cluster analysis consisted of two clusters. The average silhouette of this solution was 0.25 which means that there was no substantial structure in the data. The Fc(U) with .50 was low and the Dc(U) with also .50 was relatively high and both again indicated a high fuzziness in our data. In this second order analysis, fuzzy clustering was not able to form patient groups that are interpretable on a statistical or clinical level.

## Discussion

In our study, clinical data of patients with chest pain had an underlying structure on variable level but not on individual patient level. As a result, patients could not be grouped into clinically meaningful clusters by multivariate analyses and therefore no validation against patients' final diagnoses was possible.

A multiple correspondence analysis (MCA) supported our data collection strategy on variable level. It showed that relevant aspects were captured by the case report form. Six factors emerged from this analysis: „chest wall syndrome“, „vital threat“, „stomach and bowel pain“, „angina pectoris“, „chest infection syndrome“, and „self-limiting chest pain“. Therefore, the clinical category chest pain had a meaningful underlying structure. MCA supported our measurement strategy.

However, k-means and fuzzy cluster analysis on patient level were not able to find distinct groups. The resulting cluster solutions were not interpretable and had insufficient statistical quality criteria. The absence of any pronounced cluster structure was also confirmed by multidimensional scaling (MDS) and heat map representations of the data.

Strengths of this study are the prospective design, a large and representative consecutive sample in a primary care setting not distorted by referral patterns, and small drop out rates. Study procedures, including random audits, reduced the possibility of selection bias, and an interdisciplinary team provided a precise diagnosis as reference standard.

Individual configurations of patients on our variables were widespread with no typical groupings. This conclusion is supported by Eslick [6] who performed a k-means cluster analysis on variable level to determine whether there are characteristic patterns in chest pain locations described by patients. He found four different chest pain locations (upper chest, central retrosternal, central chest, left chest, and left arm). These groupings on variable level were not helpful in differentiating between patients with an acute coronary syndrome (ACS) and patients with non-cardiac chest pain. He concluded that chest pain is a heterogeneous category with no coherent associations between different signs and symptoms. An advantage of our study, compared to Eslick, is the larger number of variables we were able to enter in our analyses.

Other studies using multivariate techniques were done with patients already given a cardiac diagnosis. Crichton and Hinde used multiple correspondence analysis to classify patients with chest pain into those with high cardiac risk and those with low cardiac risk [25]. Correct classification rates were around 80%. In this study, the additional information on cardiac risk was used and therefore no natural groupings were examined. In their latent class cluster analysis, DeVon et al. [1] found four subgroups of patients diagnosed with acute coronary syndrome, but none of these groups contained all the classic symptoms of acute coronary syndrome. The frequency of chest pain did not differentiate between the four groups. Riegel et al. [7] also found four clusters in patients with a confirmed event of acute coronary syndrome. They selected eight symptoms to form groups in a two-step cluster analysis. They described a group with typical acute coronary syndrome, a group with pain symptoms, a group with stress symptoms, and a group with diffuse symptoms. The group with the diffuse symptoms had the highest mortality so that the validity of these results seem questionable. None of the five clusters found by Ryan et al. [8] in their latent class cluster analysis of data by 1073 patients with acute myocardial infarction contained the most prominent symptoms of acute myocardial infarction that are considered typical for this disease. Lindgren et al. [9] found three clusters in elderly patients with ischemic coronary heart disease: a typical group with severe ischemic pain, a weary group with fatigue, sleep disturbance, and shortness of breath, and a group with diffuse symptoms. The patients in the weary group were the most impaired. Except for the study of Eslick [6], all other studies recruited patients with established cardiac diagnoses and used a limited number (up to 20) of classification variables. As was shown, studies with patients with established cardiac diagnoses did not find coherent symptom patterns. The study of Ryan et al. [8] has a comparable sample size to ours. They did not find a cluster of patients that included most of the typical symptoms for acute myocardial infarction. This finding corroborates our central finding in chest pain data that no typical patient clusters emerged. All the other mentioned studies have much smaller sample sizes so that the stability of their solutions might be reduced.

To our knowledge, ours is the first study to apply MCA and MDS to chest pain data and to use cluster analysis in such detail to search for patient groups in this area. We found an underlying structure of the construct chest pain on variable level but no structure on individual level. Perhaps we have not measured all relevant aspects but we consider this to be unlikely on the basis of previous studies. We even recorded subjective impressions of physicians and patients in addition to objective medical data.

## Conclusions

Chest pain seems to be a heterogeneous and multi-faceted clinical category that has no prototypical manifestations on patient level. This might be a disillusioning result because the physician is expected to construct a diagnosis on an individual level and to categorize whether the patient has a serious condition and needs special care or not. Nevertheless, it has to be emphasized that classification of patients into a diagnostic category is possible without finding natural groups in data [14]. This was demonstrated in another article of our research group [11]. Apart from a different statistical approach, several frameworks in human reasoning like case based reasoning [26], fuzzy reasoning [27], or fast and frugal heuristics [28] have been proposed to model the underlying cognitive processes in classification.

## Competing interests

The authors declare that they have no competing interests.

## Authors' contributions

OH developed the concept for data analysis, performed the statistical analyses, and drafted the manuscript. SB participated in the concept for data analyses, participated in performing the statistical analyses, and helped to draft the manuscript. EH developed the concept for data analysis, performed the statistical analyses, and helped to draft the manuscript. RS developed the concept for data analysis, performed the statistical analyses, and helped to draft the manuscript. KD developed the concept for data analysis, performed the statistical analyses, and helped to draft the manuscript. NDB participated in the study design and coordination, the rationale for the data analyses, and helped to draft the manuscript. All authors read and approved the final manuscript.

## Pre-publication history

The pre-publication history for this paper can be accessed here:

http://www.biomedcentral.com/1471-2288/11/155/prepub

## References

[B1] DeVon HA Ryan CJ Rankin SH Cooper BA Classifying subgroups of patients with symptoms of acute coronary syndromes: A cluster analysis Res Nurs Health 2010 33 5 386 397 10.1002/nur.20395 20672306 PMC3102439

[B2] Bösner S Becker A Haasenritter J Abu Hani M Keller H Sönnichsen AC Karatolios K Schaefer JR Seitz G Baum E Chest pain in primary care: Epidemiology and pre-work-up probabilities Eur J Gen Pract 2009 15 3 141 146 10.3109/13814780903329528 19883149

[B3] Bösner S Becker A Abu Hani M Keller H Sonnichsen AC Haasenritter J Karatolios K Schaefer JR Baum E Donner-Banzhoff N Accuracy of symptoms and signs for coronary heart disease assessed in primary care Br J Gen Pract 2010 60 575 246 257 10.3399/bjgp10X502137 PMC2880766 20529488

[B4] Rutten FH Kessels AG Willems FF Hoes AW Electrocardiography in primary care; is it useful? Int J Cardiol 2000 74 2-3 199 205 10.1016/S0167-5273(00)00284-9 10962122

[B5] Gill CJ Sabin L Schmid CH Why clinicians are natural bayesians BMJ 2005 330 7499 1080 1083 10.1136/bmj.330.7499.1080 15879401 PMC557240

[B6] Eslick GD Usefulness of chest pain character and location as diagnostic indicators of an acute coronary syndrome Am J Cardiol 2005 95 10 1228 1231 10.1016/j.amjcard.2005.01.052 15877997

[B7] Riegel B Hanlon AL McKinley S Moser DK Meischke H Doering LV Davidson P Pelter MM Dracup K Differences in mortality in acute coronary syndrome symptom clusters Am Heart J 2010 159 3 392 398 10.1016/j.ahj.2010.01.003 20211300 PMC2844635

[B8] Ryan CJ DeVon HA Horne R King KB Milner K Moser DK Quinn JR Rosenfeld A Hwang SY Zerwic JJ Symptom clusters in acute myocardial infarction: a secondary data analysis Nurs Res 2007 56 2 72 81 10.1097/01.NNR.0000263968.01254.d6 17356437

[B9] Lindgren TG Fukuoka Y Rankin SH Cooper BA Carroll D Munn YL Cluster analysis of elderly cardiac patients' prehospital symptomatology Nurs Res 2008 57 1 14 23 10.1097/01.NNR.0000280654.50642.1a 18091288

[B10] Bosner S Becker A Abu Hani M Keller H Sonnichsen AC Haasenritter J Karatolios K Schaefer JR Baum E Donner-Banzhoff N Accuracy of symptoms and signs for coronary heart disease assessed in primary care Br J Gen Pract 2010 60 575 246 257 10.3399/bjgp10X502137 PMC2880766 20529488

[B11] Bosner S Haasenritter J Becker A Karatolios K Vaucher P Gencer B Herzig L Heinzel-Gutenbrunner M Schaefer JR Abu Hani M Ruling out coronary artery disease in primary care: development and validation of a simple prediction rule CMAJ 2010 182 12 1295 1300 10.1503/cmaj.100212 20603345 PMC2934794

[B12] Knottnerus JA Muris JW Assessment of the accuracy of diagnostic tests: the cross-sectional study J Clin Epidemiol 2003 56 11 1118 1128 10.1016/S0895-4356(03)00206-3 14615003

[B13] Bosner S Becker A Haasenritter J Abu Hani M Keller H Sonnichsen AC Karatolios K Schaefer JR Seitz G Baum E Chest pain in primary care: epidemiology and pre-work-up probabilities Eur J Gen Pract 2009 15 3 141 146 10.3109/13814780903329528 19883149

[B14] Kaufman L Rousseeuw PJ Finding groups in data. An introduction to cluster analysis 2005 Hoboken: Wiley

[B15] Greenacre M Blasius J Multiple Correspondence Analysis and Related Methods 2006 London: Chapman & Hall

[B16] Blasius J Korrespondenzanalyse.[Correspondence analysis] 2001 Munich: Oldenbourg

[B17] Sourial N Wolfson C Zhu B Quail J Fletcher J Karunananthan S Bandeen-Roche K Beland F Bergman H Correspondence analysis is a useful tool to uncover the relationships among categorical variables J Clin Epidemiol 2010 63 6 638 646 10.1016/j.jclinepi.2009.08.008 19896800 PMC3718710

[B18] Cox TF Cox MAA Multidimensional Scaling 2001 London: Chapman and Hall

[B19] Bacher J Pöge A Wenzig K Clusteranalyse. Anwendungsorientierte Einführung in Klassifikationsverfahren [Cluster analysis. Practical introduction in classification measures] 2010 Munich: Oldenbourg

[B20] Everitt BS Landau S Leese M Cluster analysis 2001 London: Arnold

[B21] Schendera CFG Clusteranalyse mit SPSS: Mit Faktorenanalyse. [Cluster analysis with SPSS: Including factor analysis] 2009 Munich: Oldenbourg

[B22] Romesburg CH Cluster analysis for researchers 2004 North Carolina: Lulu Press

[B23] Hintze JL NCSS User's guide IV 2007 Kaysville, Utah: NCSS

[B24] Jaccard J Becker MA Statistics for the behavioral sciences 2009 Belmont: Wadsworth

[B25] Crichton NJ Hinde JP Correspondence analysis as a screening method for indicants for clinical diagnosis Stat Med 1989 8 11 1351 1362 10.1002/sim.4780081107 2609046

[B26] Salem ABM Case based reasoning technology for medical diagnosis World Academy of Science, Engineering and Technology 2007 31 9 13

[B27] Seising R From vagueness in medical thought to the foundations of fuzzy reasoning in medical diagnosis Artif Intell Med 2006 38 3 237 256 10.1016/j.artmed.2006.06.004 16956755

[B28] Gigerenzer G Todd P Group TAR Simple heuristics that make us smart 1999 Oxford: Oxford University Press

